# Supplementary material for: Machine learning evaluation for identification of M-proteins in human serum
Source: PLoS One. 2024 Apr 2;19(4):e0299600. doi: 10.1371/journal.pone.0299600 (PMC10986985; doi:10.1371/journal.pone.0299600)
Supplement: Attachment — Submitted filename: Reviewing PONE.pdf [file pone.0299600.s006.pdf]

Summary:

The authors performed capillary & gel electrophoresis and immunofixation for more to 60k serum samples. Based on their data, they classified their samples into either “absence of M-protein” or “presence of M-protein”, and classified the subtype of M-protein, when relevant. Next, they trained various ML classifiers to automate those classification tasks, by feeding those with raw numerical data of the SPEP (Serum Protein Electrophoresis) curves, i.e. the value of each point of the SPEP curve.

The authors present an extremely valuable dataset: more than 60k samples, annotated thanks to three biological assays: capillary SPEP, gel SPEP and immunofixation; with the interpretation from laboratory experts. This alone is a solid argument supporting the publication of their results. It should be noted that the data might be described better, for instance by 1) indicating the size of M-spikes in their curves and the proportion of small/medium/large M-spikes, for instance; and 2) the proportion of other abnormal patterns which may mask or be confounded with M-proteins (artifacts (fibrinogen, iodinated contrast agents…), beta-gamma bridging). Furthermore, it is not clear if all samples were analyzed with all 3 assays: capillary SPEP, gel SPEP and immunofixation. A table may be useful here. Finally, the choice of the authors to partition their data into a training and test sets only, without any validation set, despite their number of samples, is at major risk of data leakage: there is an urge in addressing or at least discussing this point in the manuscript.

The main downside of this study, in my opinion, is the machine learning methodology used here, which is far from state of the art standards. SPEP data, is, by definition, signal data. Multiple works have highlighted the superiority of DL (deep learning) methods, mostly but not limited to CNN & transformers, to such data. However, the word “signal” is never used throughout the manuscript and DL models are absent from the models trained by the authors. The question of why the authors chose to elude this major point stays unanswered at the end of this manuscript. The ML (meachine learning) knowledge of the authors does not seem to be an obstacle to the use of DL models, based on the expertise they demonstrate in ML and DL, largely discussing the upsides and downsides of various ML models including DL ones. Furthermore, training/inference speed should not be an issue either, since 60k samples times 300 points per curve is a relatively small amount of data to process (the authors state that 20 minutes were needed to train all 26 models in this study).

The use of SHAP values, here, further highlights this problem. The models seem to simply “look at high values” in the beta & gamma regions and deduce if there is an M-spike in the sample. However, M-spikes are not always characterized by high values, and small M-spikes may only be visually detected, by the expert, thanks to an abnormal qualitative pattern, rather than by observing high quantities in the beta/gamma fractions (e.g., shoulder in the beta fraction). This is only possible with ML if treating the data as a signal, e.g., by using convolutional layers. Since the authors do not inform about the exact patterns observed in their dataset (see my previous paragraph about data), it is impossible to predict their models’ expected behavior on such samples. Unfortunately, those samples are crucial, and highly responsible for the fact that SPEP is not yet fully automated in modern laboratories.

Finally, the authors imply that their methodology may have other applications. This is highly doubtful, as they are few examples of biological assays outputting highly standardized/aligned signal data, which may give such robust results with the methodology they use. Indeed, the previous studies the authors cite to support their work have been using this kind of ML tools to make predictions based on concentrations deducted from signal data, rather than the raw data themselves.

In total, this study seems highly promising, thanks notably to a highly valuable dataset, but which should be analyzed using state of the art machine learning tools to be considered in today’s literature.

<u>Please find below the list of my comments :</u>

1-MINOR) Page 3, introduction:

- Authors should consider the use of abbreviations “SPEP” and “UPEP”, which are commonly used in related literature to designate Serum/Urine Protein Electrophoresis, instead of their proposed “S-electrophoresis” and “U-electrophoresis”. Furthermore, those latter are never used throughout the manuscript, and multiple occurences of “protein electrophoresis/es” may be replaced by their acronym in order to make the text lighter.

2-MINOR) Page 4, introduction: "haematological malignant diseases such as myeloma as well as the less severe disease monoclonal gammopathy of undetermined significance (MGUS)."

- This sentence lacks scientific accuracy, since MGUS is not a malignancy and is by definition asymptomatic rather than "less severe". Please consider rephrasing.

3-MINOR) Pages 4-5, introduction: From "Recent studies show that machine learning can be used to assess protein electrophoresis by examining protein glycan patterns to follow up tumor surgery (1)." To "extreme gradient boosted trees or extra trees classifier, provide a 95% accuracy in the assessment of plasma amino acids (2)."

- I believe that those previous works do not support the legitimacy to the authors' present study. Indeed, in those studies, ML classifiers including tree classifiers were used to analyze absolute or relative concentrations of specific molecules, i.e. N-glycans or amino-acids. Thus, in their dataset, each variable corresponded to a specific molecule. This is the right way to perform such a study.
- In their work, the authors feed their models with raw CZE (capillary zone electrophoresis) curves, which is a signal. Here, each point of the curve is not related to a specific molecule. For instance, point 120 could be in the alpha-1-globulins or in the alpha-2-globulins, and may be affected mainly by the serum concentration of haptoglobin in one sample, or by alpha-2-macroglobulin in another sample. This, to my knowledge, is not a typical use of such tree classifiers, and this methodology is not supported by the references. Consider citing works that use tree classifiers on signal data rather (or use DL models instead).

4-MINOR) Page 5, introduction: "Furthermore, artificial intelligence (AI) is used in nuclear medicine and radiology for assessment of Positron emission tomography-computed tomography PET-CT or PET/CT images from 100 different organs (3; 4). Assessment using the AI-based analysis tool Recomia showed an accuracy coefficient of 0.93 (4).".

- I do not see in which way those references are related with the present study.

5-MINOR) Page 5, introduction: « Thus, in this study, we investigated and compared several different decision tree algorithms for the detection of M-protein in 67,073 serum samples from 34,567 patients. »

- There is a high lack of scientific accuracy here, because tree classifiers cannot "analyze serum samples". The rest of the manuscript progressively uncovers what was performed exactly, but I think it would be better to clarify at this stage that data were not treated as signal, and that models were fed with the 300 raw values of the SPEP curves.

6-MINOR) Page 5, Material and Methods:

- Did the authors follow any recommendations for reporting diagnostics study, such as STARD?

7-MINOR) Page 6, Material and Methods:

- The number of samples and patients should be stated in the Results instead of the Material and Methods section. Only the dates should be cited here.

8-MINOR) Page 6, Material and Methods: "All samples were analyzed on both an automated capillary electrophoresis system (SEBIA, Issy-les-Moulineaux, France), for quantification, as well as by a semi-automated agarose gel technology system (Hydrasys, SEBIA, Issy-les-Moulineaux, France) (5; 6) to not miss small fractions of M-protein."

- "(SEBIA, Issy-les-Moulineaux, France)" should be replaced by "(Capillarys XXX, SEBIA, Issy-les-Moulineaux, France)", to keep consistency with the following "(Hydrasys, SEBIA, Issy-les-Moulineaux, France)". "Capillarys XXX" should be replaced by the model used (Capillarys 2 Flex Piercing, 3 Octa, 3 Tera…?)
- "agarose gel technology system (Hydrasis)" is unclear, since the Hydrasis may perform both serum electrophoresis and serum immunofixation, which both use a gel electrophoresis system. I believe the authors refer to serum gel electrophoresis, but it would be clearer to explicitly specify this.
- It is unclear if the authors analyzed all samples by both capillary zone electrophoresis (CZE) (Capillarys) and gel electrophoresis; or if gel electrophoresis was used only for samples analyzed by CZE which displayed minor abnormal patterns. This paragraph

implies that both techniques were used for all samples, but the lack of automation of the gel electrophoresis method makes it highly unlikely that 60k gel electrophoreses were performed between 2015 and 2020.

- If only a subset of all samples were analyzed by gel electrophoresis, the number should be stated (in the Results section)

9-MINOR) Page 6, Material and Methods: "M-proteins were quantified based on the capillary electrophoresis (CE) electropherogram curve".

- The authors may use the abbreviation CZE for capillary ***zone*** electrophoresis, which is more widely used in relevant literature.

10-MINOR) Page 6, Material and Methods: "using the Sebia software (SEBIA, Issy-lesMoulineaux, France)"

- The name of this software (Phoresis) should be stated.

11- MINOR) Page 6, Material and Methods, *Data processing*: "For the training and classification of M-proteins we used the labeled anonymized dataset described above, consisting of a total of 67,073 samples."

- As discussed earlier, the number of extracted samples should be stated in the Results section, not the Material and Methods section.

12- MINOR) Page 6, Material and Methods, *Data processing*: "All data were assessed by medical experts who determined whether M-protein was present or not based on electropherograms from capillary gel electrophoresis"

- I believe the authors mean "capillary ***OR*** gel electrophoresis" here?

13-MAJOR) Page 6, Material and Methods, *Data processing*: "The data was randomly divided into 70% for training and 30% for testing."

- The authors state that they have partitioned their data into a training set and a test set. However, the methodology they describe is as follow: 1) train each model; 2) compare models between each other using the described metrics; 3) test results on new data.

Those three steps are usually performed using three datasets, namely training, validation and test set, respectively. Using training data to perform step 2 would lead to potentially selected the most overfitted model (which is obviously useless); and using test data to perform step 2 would be described as data leakage. I believe this is a point here that should be extensively discussed by the authors, or better described if my interpretation is incorrect.

14-MINOR / Page 6, Material and Methods, *Data processing*: "Feature importance was based on the shapely values from the shapely 1.7.1 library."

- Replace "shapely" by "Shapley".

15-MINOR / Page 7-8, Material and Methods, sections *"Extra Trees regressor and Random Forest regressor settings"*, *"Histogram Gradient Boosting regressor settings"*, *"Light Gradient Boosting regressor settings"*, *"Extreme Gradient Boosting regressor settings"*

- Those sections weigh down the manuscript and would benefit from being moved in a Supplementary Material and Methods file.

16-MINOR / Page 8, Results: "For this study we used a total of 67,073 patient serum samples of which 14,626 were positive for M-protein."

- It would be interesting if the authors indicated the percentage of the different abnormal patterns observed in their data. For instance:
    - 1) what range of M-protein concentration did they observe (e.g. give quantiles)? Detecting small M-spikes (< 1g/L, or even <0,5g/L which is announced by SEBIA as the limit of quantification with the CE method) is more difficult on CE than large M-spikes.
    - 2) what different abnormal patterns were observed among the samples? Patterns such as restricted heterogeneity of gammaglobulins without clear M-spike, oligoclonal bands or beta-gamma bridging are complex patterns that may be confounded with M-spikes and it would be interesting to know their proportion in the dataset.

17-MINOR / Page 8, Results: "The patient demographics and distribution of monoclonal isotypes are shown in Figure 1A and 1B".

- Figure 1A-left is a table with only 3 values; those should be directly stated in the text rather than in a Figure.
- I do not understand relevance of Figure 1A-right. If I understand correctly, those are the quartiles of age after separating patients by age; however it does not allow knowing how many patients are in each age group (i.e. 0-25, 26-50, 51-75, 76+)?
- Figure 1B-left is a table, and thus should be presented as a table, to avoid formatting inconsistencies with other tables in the final manuscript.

18-MAJOR / Page 8, Results:

- Figure 1B-left (table) indicates that 1,962 samples had M-protein of unspecified nature. How is it possible? Were those possibly artifacts, such as antibiotic drugs, iodinated contrast products, fibrinogen…? Were those samples for which immunofixation was not performed despite current recommendations?
- Figure 1B-left (table) indicates that only 3 samples were found with isolated free light chains. This seems a very low incidence; maybe the authors should discuss this prevalence in regard to existing MGUS/myeloma literature? Since samples with small M-spikes and/or free light chains are, even with human analysis, the hardest to classify, this prevalence may have an influence of the authors' final results.

19-MINOR / Page 9, Results, Decision tree methods detect M-proteins with high accuracy:

- All results presented in Table 1 are also presented in this section, which 1) creates redundancy and 2) burdens the text. Consider either removing Table 1 or keeping things simple in the main text.
- Furthermore, it would be highly interesting to see detailed results for some particular patterns. For instance, do the models display consistent accuracy when tested on samples with small M-spikes (e.g., < 1 g/L), and/or for samples with isolated free light chains?

20-MINOR / Page 10, Results, M-protein detection by decision tree methods can be verified to the gamma and beta fractions: "A successful outcome was one which, for our application, correctly predicted the M-protein"

- If I understand correctly, "predicted the M-protein" should be replaced by "predicted the presence of an M-protein" here, to distinguish from the task of M-protein classification, which is discussed later.

21-MINOR / Page 10, Results, M-protein detection by decision tree methods can be verified to the gamma and beta fractions: "The results verified the gamma globulin fraction and part of the beta-2 globulin fraction as the most important for detecting M-protein (Fig. 3A, Suppl Fig. 2), corresponding to the features with migration times between 251 and 260 seconds in the electropherogram (Fig. 3B), thereby verifying the results and further strengthening the reliability of the top five algorithms."

- The use of SHAP here, like the use of tree classifiers for the analysis of signal, seems highly irrelevant as discussed earlier. Since tree classifiers do not "see" patterns (like convolutional NN with a signal input) but only independent values, M-spikes may only be detected from such input data by high absolute values for points involved in the M-spike. At the very least, the authors may have used this SHAP methodology to seek if points with high SHAP values corresponded to points in the M-spike (or if the annotation is not available, to points with high absolute values = concentration), per sample.

22-MINOR / Page 10, Results, M-protein detection by decision tree methods can be verified to the gamma and beta fractions: "Furthermore, using SHAP values also enabled us to compute an M-protein probability score for each patient (Fig. 3C), which can be used to predict the likelihood of M-protein being present for a specific patient"

- What would be the difference with predicting the probability instead of the label, using the sklearn package?

23-MAJOR / Pages 10-11, Extra Trees and Random Forest show the best success rate in classifying M-protein isotypes: "For this purpose, we had to exclude the samples with free light chain Mprotein, IgD M-protein, and samples with more than one type of M-protein in our

data set, since these samples reached less than 1% of the total M-protein positive samples and were too few to be properly trained and tested by the algorithms."

- I understand the decision of the authors to keep negative samples (i.e., without M-protein) in the training set to keep a robust sample size and increase the trainability of their models. Even though I think it would have been best to train those models with only positive samples (i.e., a dual model pipeline, the first detecting a M-protein and the second classifying it) in order to maintain a high specialization of each model (use the power of two weaker classifiers to make a strong & robust ensemble classifier).
- However, I believe that presenting results including those normal=negative samples leads to biased metrics, since negative samples are the most represented samples and at the same time the easiest to classify (no M-protein = no risk of misclassifying the M-protein).

24-MAJOR / Page 11, M-protein detection by decision tree methods can be verified to the gamma and beta fractions:

- The authors show that their models quite accurately classify IgG, less accurately IgA, and even less accurately IgM.
- This would be expected by a "random guest" from an expert, since: 1) IgG are the most common, and commonly found in gammaglobulins; 2) IgM also elude in the gammaglobulins fraction, but are heavier than IgG, so usually responsible of taller M-spikes in the gammaglobulins fraction; 3) IgA are more rare than IgG, but most commonly found in betaglobulins
- How do the authors compare their results to such a "random guest"? For instance, it would be possible to
  - 1) make a simple expert system that would systematically classify betaglobulins M-spikes as IgA, and gammaglobulins spikes as IgG or IgM according to the size of the M-spike (by using a threshold determined using the training set)
  - 2) compare the trained models to this simple expert system to see if the proposed method outperforms it
- At the very least, this point should be addressed in the Discussion section.

25-MINOR / Page 11, Discussion: "Analysis of fractionated serum proteins by electrophoresis is the gold standard to evaluate acute phase reactions and immunoglobulin patterns in the individual to help form a diagnosis."

- Consider revising this sentence which is not entirely accurate: 1) SPEP is a screening method rather than a diagnosis method, which necessitates a confirmation by another technique such as immunofixation; which is a gold standard technique; and 2) acute phase reactions' gold standard technique would more likely be considered to be plasma CRP, to my knowledge.

26-MINOR / Page 11, Discussion: "due to excessive amounts of M-protein"

- Remove "excessive amounts" (the sole presence of a M-protein is, by definition, pathological even without clinical significance)

27-MINOR / Page 11, Discussion:

- References 1, 3 and 4 are, in my opinion, inappropriate here, as already discussed earlier.

28-MINOR / Page 11, Discussion: "The discrepancy in accuracy score between our model and the model by Chabrun et al is likely attributable to the fact that different methods were used, but also highlights issues that can arise for networks of high complex nature"

- I believe the origin of this difference is most likely to be attributable to the fact that Chabrun et al. had less precise annotations, since they only performed SPEP. Thus, some samples were classified as M-spike, while some were classified as "restricted heterogeneity of gammaglobulins" (i.e., suspicion of abnormal pattern without clearly defined M-spike). They have, in their study, highlighted than even human experts do not clearly define and agree, for some samples, between normal samples, restricted heterogeneity of gammaglobulins and M-spikes.
- Here, the authors present extremely robust results, which, I believe, were obtained at least in part thanks to clearly defined, binary labels, which were possible thanks to the use of multiple assays (capillary SPEP, gel SPEP, immunofixation). I think the authors should better emphasize this, as it add significant value to their work compare to previous works in the field.

- However, I think that the sentence “but also highlights issues that can arise for networks of high complex nature” should be discarded: in this context, it has no supportive arguments, and is more likely to reflect the authors’ preference for tree classifiers rather than a solid argument towards the performance of those models compared to ANN/CNN, since those have shown far superior performance in many applications found in literature.

29-MINOR / Page 13, Discussion: “The use of decision tree methods can be more transparent compared to deep learning algorithms, if implemented as we propose here”

- I think there is too few arguments supporting the fact that tree classifiers (particularly extremely complex models such as the ones trained here) are more transparent than DL classifiers here. The use of the SHAP values seems to be the most recurring argument, however it is a model-agnostic method and thus applicable to ANN/CNN similarly.
