# Supplementary material for: Machine learning evaluation for identification of M-proteins in human serum
Source: PLoS One. 2024 Apr 2;19(4):e0299600. doi: 10.1371/journal.pone.0299600 (PMC10986985; doi:10.1371/journal.pone.0299600)
Supplement: Attachment — Submitted filename: Response to Reviewers.pdf [file pone.0299600.s007.pdf]

We thank the Reviewers for their constructive and insightful comments and suggestions, which we believe have considerably improved our manuscript. We have fully addressed the reviewers' comments and concerns in a point-by-point response below (highlighted in blue). All changes made to the manuscript are shown with track changes in the "Revised Article with Changes Highlighted" file. We hope that the reviewing process finds our revised manuscript acceptable for publication in *PLOS ONE*.

Reviewer #1

Summary:

The authors performed capillary & gel electrophoresis and immunofixation for more to 60k serum samples. Based on their data, they classified their samples into either "absence of M-protein" or "presence of M-protein", and classified the subtype of M-protein, when relevant. Next, they trained various ML classifiers to automate those classification tasks, by feeding those with raw numerical data of the SPEP (Serum Protein Electrophoresis) curves, i.e. the value of each point of the SPEP curve.

The authors present an extremely valuable dataset: more than 60k samples, annotated thanks to three biological assays: capillary SPEP, gel SPEP and immunofixation; with the interpretation from laboratory experts. This alone is a solid argument supporting the publication of their results. It should be noted that the data might be described better, for instance by

1) indicating the size of M-spikes in their curves and the proportion of small/medium/large M-spikes, for instance

We thank the reviewer for the suggestion, and we have now added a table (new Table 2) to show the distribution of small and large M-protein fractions (for IgG, IgA and IgM) in our data set, divided into tertiles: <1 g/l, 1-5 g/l and >5 g/l. In addition, we have also included a figure in the supplementary material (new supplementary figure S2), showing the specific distribution of free light chain concentrations in the data set (see our comments on revised data of FLC-positive samples under point 18 below).

2) the proportion of other abnormal patterns which may mask or be confounded with M-proteins (artifacts (fibrinogen, iodinated contrast agents…), beta-gamma bridging).

We agree with the reviewer that it would be very valuable with more information regarding abnormal patterns other than the presence of M-proteins, which could influence the interpretation of the results. Unfortunately, we don't have any practical possibility or resources to screen or search the 67,000 + samples in data set for such information. However, we have now added this in the Discussion section text as a possible limiting factor (page 20 in the revised manuscript). Of note, we use serum samples and only very rarely do we analyze plasma samples for detection of M-proteins, so fibrinogen artifacts are not expected to be a factor that influences the results. Iodinated contrast agent artifacts are also very rarely seen since we have very few patients that have been exposed to intravenous contrast at x-ray examination in relation to the blood sample being taken.

Furthermore, it is not clear if all samples were analyzed with all 3 assays: capillary SPEP, gel SPEP and immunofixation. A table may be useful here.

All samples were analyzed by both capillary SPEP and gel SPEP (pentascreen). Immunofixation analysis was only performed when a suspected M-protein was present (based on the results from the capillary SPEP and gel SPEP). We have now clarified this better in the Materials and Methods section.

Finally, the choice of the authors to partition their data into a training and test sets only, without any validation set, despite their number of samples, is at major risk of data leakage: there is an urge in addressing or at least discussing this point in the manuscript.

We agree with the Referee in regards to the importance of validation sets in ensuring robust model generalization. The reason for not including such a set in the current study is related to the lack of a sufficiently balanced and representative data set for all classes of M-proteins. Our data set comprises diverse M-protein isotypes, some of which have limited representation due to their rarity in the clinical data set we received. As a result, creating a validation set that accurately represents the underlying distribution of classes would introduce large imbalances during training and testing. We therefore made a conscious decision to prioritize maintaining data integrity and preventing further biases due to imbalanced class representation. That was actually another reason for choosing a decision tree approach since, unlike deep learning networks, decision trees are better at handling imbalances in data (they can learn to prioritize minority classes since the impurity-based splitting criteria, such as the Gini index or entropy, are sensitive to class imbalances). Finally, we should point out that the test set is not included or used in any way during training. Our trees are taught solely on the training set. Typically, the test set would be used to adjust and improve decision trees by manipulating their hyper-parameters. As stated in the manuscript, we used the default values of all hyper-parameters. Thus, there is no risk of data leakage since the test set is not used during training.

We have now tried to clarify this better in the manuscript in both the materials and methods section as well as in the discussion.

The main downside of this study, in my opinion, is the machine learning methodology used here, which is far from state of the art standards. SPEP data, is, by definition, signal data. Multiple works have highlighted the superiority of DL (deep learning) methods, mostly but not limited to CNN & transformers, to such data. However, the word "signal" is never used throughout the manuscript and DL models are absent from the models trained by the authors. The question of why the authors chose to elude this major point stays unanswered at the end of this manuscript. The ML (meachine learning) knowledge of the authors does not seem to be an obstacle to the use of DL models, based on the expertise they demonstrate in ML and DL, largely discussing the upsides and downsides of various ML models including DL ones. Furthermore, training/inference speed should not be an issue either, since 60k samples times 300 points per curve is a relatively small amount of data to process (the authors state that 20 minutes were needed to train all 26 models in this study).

Indeed, while deep learning models, including CNNs and transformers, have shown great success in processing and analyzing images and signal data, we believe that they would not be as successful for our signal data. Our data comprises of short input sequences (300 numerical values per sample) which can pose challenges for deep networks with convolutions due to the limited context they provide. To be more specific, it is indeed possible to try passing our data to deep networks as follows: we could process the signals to form matrices (in other words creating "images" from these numbers) but then their size would shrink to a tile of size of just 17x18 pixels (this is even smaller than the MNIST dataset). We felt this was too small to train CNNs with and certainly too small for transformers which are known to require extensive training data for any possibility of success. We also agree with the Referee that a DL approach would be better but in that respect we would need to find a better pre-processing format of the signal data for it to be successful. Instead, we are certain these CNNs or transformer networks would be much more appropriate to apply to digital gel images, but we don't have such images in our data set. Deep learning models tend to excel when there is a significant amount of data (such as gel images for instance) available for them to learn meaningful hierarchical representations and patterns. In cases where the input sequences are short, such as ours, there might be a risk of overfitting or the models struggling to capture meaningful patterns effectively. Finally, as also mentioned above, since our data was also imbalanced there was a concern that a DL approach would not fair as well as a decision tree approach.

We have added a paragraph in the discussion section to better clarify our choice of model.

The use of SHAP values, here, further highlights this problem. The models seem to simply "look at high values" in the beta & gamma regions and deduce if there is an M-spike in the sample. However, M-spikes are not always characterized by high values, and small M-spikes may only be visually detected, by the expert, thanks to an abnormal qualitative pattern, rather than by observing high quantities in the beta/gamma fractions (e.g., shoulder in the beta fraction). This is only possible with ML if treating the data as a signal, e.g., by using convolutional layers. Since the authors do not inform about the exact patterns observed in their dataset (see my previous paragraph about data), it is impossible to predict their models' expected behavior on such samples. Unfortunately, those samples are crucial, and highly responsible for the fact that SPEP is not yet fully automated in modern laboratories.

We acknowledge the Referee observation that the use of SHAP values may give the impression that our models focus solely on high values in the beta and gamma regions. We want to clarify that we only displayed the most important SHAP values in our article. In general, the visualization of SHAP values may sometimes emphasize specific data points of high importance but does not reflect the entirety of our model's decision-making process. It is a fact that decision trees combine information from the full feature space to understand patterns and make decisions. We have now therefore included a new figure (S4 Fig) in the supplementary material, which illustrates how our models utilize information from a large range of the electrophoresis curve for M-protein detection. This figure demonstrates that the models, in their decision making, take into account a large part of the electrophoresis curve with different weights of importance.

Finally, the authors imply that their methodology may have other applications. This is highly doubtful, as they are few examples of biological assays outputting highly standardized/aligned signal data, which may give such robust results with the methodology they use. Indeed, the previous studies the authors cite to

support their work have been using this kind of ML tools to make predictions based on concentrations deducted from signal data, rather than the raw data themselves.

In total, this study seems highly promising, thanks notably to a highly valuable dataset, but which should be analyzed using state of the art machine learning tools to be considered in today's literature.

Our comment was meant mostly in terms of studies on standardized signal data such as EKGs and EEGs which show how decision trees on such raw data can produce useful information about the patient's health. We have now included some recent studies (see point 3 below) where decision trees are showcased in analyzing standardized signal data from EKGs, EEGs and others in the introduction.

Please find below the list of my comments :

1-MINOR) Page 3, introduction:

• Authors should consider the use of abbreviations "SPEP" and "UPEP", which are commonly used in related literature to designate Serum/Urine Protein Electrophoresis, instead of their proposed "S-electrophoresis" and "U-electrophoresis". Furthermore, those latter are never used throughout the manuscript, and multiple occurences of "protein electrophoresis/es" may be replaced by their acronym in order to make the text lighter.

We have now changed to "SPEP" and "UPEP" throughout the text in the manuscript.

2-MINOR) Page 4, introduction: "haematological malignant diseases such as myeloma as well as the less severe disease monoclonal gammopathy of undetermined significance (MGUS)."

• This sentence lacks scientific accuracy, since MGUS is not a malignancy and is by definition asymptomatic rather than "less severe". Please consider rephrasing.

We have removed "less severe" and replaced by "asymptomatic".

3-MINOR) Pages 4-5, introduction: From "Recent studies show that machine learning can be used to assess protein electrophoresis by examining protein glycan patterns to follow up tumor surgery (1)." To "extreme gradient boosted trees or extra trees classifier, provide a 95% accuracy in the assessment of plasma amino acids (2)."

• I believe that those previous works do not support the legitimacy to the authors' present study. Indeed, in those studies, ML classifiers including tree classifiers were used to analyze absolute or relative concentrations of specific molecules, i.e. N-glycans or amino-acids. Thus, in their dataset, each variable corresponded to a specific molecule. This is the right way to perform such a study.

• In their work, the authors feed their models with raw CZE (capillary zone electrophoresis) curves, which is a signal. Here, each point of the curve is not related to a specific molecule. For instance, point 120 could be in the alpha-1-globulins or in the alpha-2-globulins, and may be affected mainly by the serum concentration of haptoglobin in one sample, or by alpha-2-macroglobulin in another sample. This,

to my knowledge, is not a typical use of such tree classifiers, and this methodology is not supported by the references. Consider citing works that use tree classifiers on signal data rather (or use DL models instead).

We have now added other references citing the type of studies the reviewer requested to the Introduction and Discussion sections:

1. Alarsan et al successfully classified electrocardiogram features using decision tree algorithms, where Random Forest achieved an overall accuracy of 96.75% in classifying heartbeats associated with cardiac arrhythmia.
2. Küçükakarsu et al analyzed EEG signals in hearing test processes using decision tree algorithms and found that LGBM gave the best performance followed by Random Forest.
3. Monari et al found differential serum protein patterns between non small cell lung cancer patients and healthy subjects using decision tree algorithms when evaluating protein mass spectra peak signals from Surface Enhanced Laser Desorption/Ionization Time-of-Flight Mass Spectrometry (SELDI-ToF-MS).

These studies, as well as other studies reported in the literature, support the use of decision tree algorithms for classifying raw signal data.

4-MINOR) Page 5, introduction: "Furthermore, artificial intelligence (AI) is used in nuclear medicine and radiology for assessment of Positron emission tomography-computed tomography PET-CT or PET/CT images from 100 different organs (3; 4). Assessment using the AI-based analysis tool Recomia showed an accuracy coefficient of 0.93 (4).".

• I do not see in which way those references are related with the present study.

These references were added as examples of how machine learning av different types are already being implemented in the clinic in various disciplines. We removed the references from the Discussion section, but kept them in the Introduction since we feel they are relevant there.

5-MINOR) Page 5, introduction: « Thus, in this study, we investigated and compared several different decision tree algorithms for the detection of M-protein in 67,073 serum samples from 34,567 patients. »

• There is a high lack of scientific accuracy here, because tree classifiers cannot "analyze serum samples". The rest of the manuscript progressively uncovers what was performed exactly, but I think it would be better to clarify at this stage that data were not treated as signal, and that models were fed with the 300 raw values of the SPEP curves.

We are unable to locate "analyze serum samples" in the text or similar text that states that tree classifiers can analyze serum samples. We are therefore unsure if there is a misunderstanding. However, in this paragraph we merely meant that tree classifiers can be used as a decision support for the medical doctors that perform the evaluation. We have tried to change the text according to the reviewer's request and hope that the reviewer is satisfied with these changes:

“Raw numerical signal data from the capillary SPEP was used and the different algorithms were evaluated for their detection accuracy as well as used for identification and verification of critical features in the data toward diagnosis.”

6-MINOR) Page 5, Material and Methods:

• Did the authors follow any recommendations for reporting diagnostics study, such as STARD?

We did not follow any particular protocol for reporting the study, but after the reviewer’s comment we have looked through all checkpoints for STARD (https://www.equator-network.org/wp-content/uploads/2015/03/STARD-2015-checklist.pdf) and can conclude that our work aligns very well with the STARD recommendations, particularly following the suggested changes requested by the reviewers here. We thank the reviewers for the suggestions and for improving our manuscript, and believe that the changes have made the manuscript more complete and in very good alignment with STARD.

7-MINOR) Page 6, Material and Methods:

• The number of samples and patients should be stated in the Results instead of the Material and Methods section. Only the dates should be cited here.

The number of samples and patients has been removed from the Materials and Methods section and is now only stated in the Results section as requested. The number of patients were 32,940, which is less than we stated in the original manuscript. Because of data anonymity, we have to rely on some personal data being provided to us by others. We were informed that some individuals were apparently double counted originally, but this is now corrected.

8-MINOR) Page 6, Material and Methods: “All samples were analyzed on both an automated capillary electrophoresis system (SEBIA, Issy-les-Moulineaux, France), for quantification, as well as by a semi-automated agarose gel technology system (Hydrasys, SEBIA, Issy-les-Moulineaux, France) (5; 6) to not miss small fractions of M-protein.”

• “(SEBIA, Issy-les-Moulineaux, France)” should be replaced by “(Capillarys XXX, SEBIA, Issy-les-Moulineaux, France)”, to keep consistency with the following “(Hydrasys, SEBIA, Issy-les-Moulineaux, France)”. “Capillarys XXX” should be replaced by the model used (Capillarys 2 Flex Piercing, 3 Octa, 3 Tera…?)

We have now made the changes requested.

• “agarose gel technology system (Hydrasis)” is unclear, since the Hydrasis may perform both serum electrophoresis and serum immunofixation, which both use a gel electrophoresis system. I believe the authors refer to serum gel electrophoresis, but it would be clearer to explicitly specify this.

"Serum gel electrophoresis" has been added to the text.

• It is unclear if the authors analyzed all samples by both capillary zone electrophoresis (CZE) (Capillarys) and gel electrophoresis; or if gel electrophoresis was used only for samples analyzed by CZE which displayed minor abnormal patterns. This paragraph implies that both techniques were used for all samples, but the lack of automation of the gel electrophoresis method makes it highly unlikely that 60k gel electrophoreses were performed between 2015 and 2020.

• If only a subset of all samples were analyzed by gel electrophoresis, the number should be stated (in the Results section)

All samples were analyzed by both capillary SPEP and gel SPEP (pentascreen), which is standard for all samples at our clinic. Immunofixation analysis was only performed when a suspected M-protein was present (based on the results from the capillary SPEP and gel SPEP). We have now clarified this better in the materials and methods section.

9-MINOR) Page 6, Material and Methods: "M-proteins were quantified based on the capillary electrophoresis (CE) electropherogram curve".

• The authors may use the abbreviation CZE for capillary zone electrophoresis, which is more widely used in relevant literature.

We have now changed to "CZE" throughout the manuscript.

10-MINOR) Page 6, Material and Methods: "using the Sebia software (SEBIA, Issy-lesMoulineaux, France)"

• The name of this software (Phoresis) should be stated.

"Phoresis" has been added.

11- MINOR) Page 6, Material and Methods, Data processing: "For the training and classification of M-proteins we used the labeled anonymized dataset described above, consisting of a total of 67,073 samples."

• As discussed earlier, the number of extracted samples should be stated in the Results section, not the Material and Methods section.

We have removed the number of samples from Materials and Methods.

12- MINOR) Page 6, Material and Methods, Data processing: “All data were assessed by medical experts who determined whether M-protein was present or not based on electropherograms from capillary gel electrophoresis”

• I believe the authors mean “capillary OR gel electrophoresis” here?

Actually, we mean capillary AND gel electrophoresis, since all samples are analyzed by both. We have now made the appropriate change in the text.

13-MAJOR) Page 6, Material and Methods, Data processing: “The data was randomly divided into 70% for training and 30% for testing.”

• The authors state that they have partitioned their data into a training set and a test set. However, the methodology they describe is as follow: 1) train each model; 2) compare models between each other using the described metrics; 3) test results on new data.

Those three steps are usually performed using three datasets, namely training, validation and test set, respectively. Using training data to perform step 2 would lead to potentially selected the most overfitted model (which is obviously useless); and using test data to perform step 2 would be described as data leakage. I believe this is a point here that should be extensively discussed by the authors, or better described if my interpretation is incorrect.

Part of this comment was addressed in our reply above. We would also like to clarify here that the test data set is never used during the training step in order to precisely avoid such effects as data leakage. It would be incorrect to do so and overall this would lead to poor generalization performance which would render the model useless in other applications. The test set is not used to improve any of the model hyper-parameters since we use the default values for each model. Also, in step 2, we indeed use the top-best of those models found during training in order to fully train them later with the full data. If these models were overfitted we would observe that the accuracy is reduced during testing. Please also note that the Extreme Gradient Boosting regressor used in our study is outfitted with mechanisms which prevent overfitting. Based on the metrics presented (on the unseen test data) we do not observe overfitting effects. This is however a point which indeed was not sufficiently addressed in the manuscript and we now clarify this by adding the following text in the Materials and Methods section:

"During training, only the training set is used to exclusively train the models, avoiding exposure to the test set. This precaution guards against overfitting as well as data leakage, preserving models' generalization ability. We also note that some of the models trained, such as for instance XGB, incorporate mechanisms to mitigate overfitting, ensuring robust generalization."

14-MINOR / Page 6, Material and Methods, Data processing: “Feature importance was based on the shapely values from the shapely 1.7.1 library.”

• Replace “shapely” by “Shapley”.

We have now changed to “Shapely”.

15-MINOR / Page 7-8, Material and Methods, sections “Extra Trees regressor and Random Forest regressor settings”, “Histogram Gradient Boosting regressor settings”, “Light Gradient Boosting regressor settings”, “Extreme Gradient Boosting regressor settings”

• Those sections weigh down the manuscript and would benefit from being moved in a Supplementary Material and Methods file.

We have now moved the description of the decision tree settings to a supplementary Material and Methods section as requested.

16-MINOR / Page 8, Results: “For this study we used a total of 67,073 patient serum samples of which 14,626 were positive for M-protein.”

• It would be interesting if the authors indicated the percentage of the different abnormal patterns observed in their data. For instance:

o 1) what range of M-protein concentration did they observe (e.g. give quantiles)? Detecting small M-spikes (< 1g/L, or even <0,5g/L which is announced by SEBIA as the limit of quantification with the CE method) is more difficult on CE than large M-spikes.

We thank the reviewer for the suggestion, and we have now added a table (new Table 2) to show the distribution of small and large M-protein fractions (for IgG, IgA and IgM) in our data set, divided into tertiles: <1 g/l, 1-5 g/l and >5 g/l. In addition, we have also included a figure in the supplementary material (new supplementary figure S2), showing the specific distribution of free light chain concentrations in the data set (see our comments on revised data of FLC-positive samples under point 18 below).

o 2) what different abnormal patterns were observed among the samples? Patterns such as restricted heterogeneity of gammaglobulins without clear M-spike, oligoclonal bands or beta-gamma bridging are complex patterns that may be confounded with M-spikes and it would be interesting to know their proportion in the dataset.

We agree with the reviewer that it would be very valuable with more information regarding abnormal patterns other than the presence of M-proteins, which could influence the interpretation of the results. Unfortunately, we don’t have any practical possibility or resources to screen or search the 67,000 + samples in data set for such information. However, we have now added this in the Discussion section text as a possible limiting factor (page 20 in the revised manuscript).

17-MINOR / Page 8, Results: “The patient demographics and distribution of monoclonal isotypes are shown in Figure 1A and 1B”.

• Figure 1A-left is a table with only 3 values; those should be directly stated in the text rather than in a Figure.

Left table is now removed and the values are incorporated in the text instead as requested.

• I do not understand relevance of Figure 1A-right. If I understand correctly, those are the quartiles of age after separating patients by age; however it does not allow knowing how many patients are in each age group (i.e. 0-25, 26-50, 51-75, 76+)?

The idea with this figure was to show the age mean and distribution within each age quartile. We have now added the number of individuals as well for each age quartile, shown in the figure inset.

• Figure 1B-left is a table, and thus should be presented as a table, to avoid formatting inconsistencies with other tables in the final manuscript.

The table in Figure 1B has now been removed from the figure and added as a separate table (new Table 1). The text has been changed accordingly.

18-MAJOR / Page 8, Results:

• Figure 1B-left (table) indicates that 1,962 samples had M-protein of unspecified nature. How is it possible? Were those possibly artifacts, such as antibiotic drugs, iodinated contrast products, fibrinogen…? Were those samples for which immunofixation was not performed despite current recommendations?

Those were samples that were confirmed to have an M-protein present, but the description in the data set file did not specify which isotype of M-protein was detected for those particular samples. However, we have now gone over the data for these samples again and were able to extract the specific type of M-protein for each sample. We have changed Figure 1B and (new) Table 1 accordingly and included these samples in the re-run of the algorithms for the detection of the specific M-protein types (new Tables 4 and 5).

• Figure 1B-left (table) indicates that only 3 samples were found with isolated free light chains. This seems a very low incidence; maybe the authors should discuss this prevalence in regard to existing MGUS/myeloma literature? Since samples with small M-spikes and/or free light chains are, even with human analysis, the hardest to classify, this prevalence may have an influence of the authors' final results.

We thank the reviewer for this very important observation. Our data set includes two different types of annotations for FLC-positive samples. This was missed when creating Figure 1B, and only one type of annotation was used when searching for FLC-positive samples. When including all samples with FLC (i.e. both types of annotations), the number of FLC-positive samples are in total 1,077, of which 404 are samples with FLC M-protein alone, and the remaining 673 samples are samples with FLC M-protein in combination with a heavy chain M-protein. We have now corrected Figure 1B (and the corresponding Table 1), changed the results text accordingly, as well as included figures of the concentration range of FLC-positive samples in the supplementary data (new Supplementary Figure S2). It is important to note that even though the number of FLC-positive samples are larger than first reported, they are

unfortunately still too few to use for training and testing of the algorithm for detecting the FLC type of M-protein. The proportion of samples containing FLC M-protein is still less than the expected proportion of about 15% reported in the literature. We have included a comment of this in the discussion section (limiting factors, page 20).

19-MINOR / Page 9, Results, Decision tree methods detect M-proteins with high accuracy:

• All results presented in Table 1 are also presented in this section, which 1) creates redundancy and 2) burdens the text. Consider either removing Table 1 or keeping things simple in the main text.

We have kept the table (new Table 3) and simplified the text as requested.

• Furthermore, it would be highly interesting to see detailed results for some particular patterns. For instance, do the models display consistent accuracy when tested on samples with small M-spikes (e.g., < 1 g/L), and/or for samples with isolated free light chains?

No, the accuracy is not consistent for any of the models for M-spikes of different sizes. The scoring for small M-spikes is much poorer than larger ones as expected, see the table below on the scoring for different sizes of M-spikes for the HGBR algorithm. It is hard to evaluate the models in this context since we have so few samples in the small size categories (see new Table 2). It would be expected that the models score poorer for small M-spikes, but to what degree is impossible to evaluate with the low number of samples we have in the small size category in our data set.

HGBR

**M-protein concentration**	**<1 g/l**	**1-5 g/l**	**>5 g/l**
F1 score for IgG	0	0.66	0.98
F1 score for IgA	0	0.21	0.80
F1 score for IgM	0	0.25	0.69

20-MINOR / Page 10, Results, M-protein detection by decision tree methods can be verified to the gamma and beta fractions: "A successful outcome was one which, for our application, correctly predicted the M-protein"

• If I understand correctly, "predicted the M-protein" should be replaced by "predicted the presence of an M-protein" here, to distinguish from the task of M-protein classification, which is discussed later.

The text has been changed to "predicted the presence of an M-protein".

21-MINOR / Page 10, Results, M-protein detection by decision tree methods can be verified to the gamma and beta fractions: "The results verified the gamma globulin fraction and part of the beta-2 globulin fraction as the most important for detecting M-protein (Fig. 3A, Suppl Fig. 2), corresponding to

the features with migration times between 251 and 260 seconds in the electropherogram (Fig. 3B), thereby verifying the results and further strengthening the reliability of the top five algorithms."

• The use of SHAP here, like the use of tree classifiers for the analysis of signal, seems highly irrelevant as discussed earlier. Since tree classifiers do not "see" patterns (like convolutional NN with a signal input) but only independent values, M-spikes may only be detected from such input data by high absolute values for points involved in the M-spike. At the very least, the authors may have used this SHAP methodology to seek if points with high SHAP values corresponded to points in the M-spike (or if the annotation is not available, to points with high absolute values = concentration), per sample.

We acknowledge the Referee observation that the use of SHAP values may give the impression that our models focus solely on high values in the beta and gamma regions. We want to clarify that we only displayed the most important SHAP values in our article. In general, the visualization of SHAP values may sometimes emphasize specific data points of high importance but does not reflect the entirety of our model's decision-making process. It is a fact that decision trees combine information from the full feature space to understand patterns and make decisions. We have now therefore included a new figure (S4 Fig) in the supplementary material, which illustrates how our models utilize information from a large range of the electrophoresis curve for M-protein detection. This figure demonstrates that the models, in their decision making, take into account a large part of the electrophoresis curve with different weights of importance.

22-MINOR / Page 10, Results, M-protein detection by decision tree methods can be verified to the gamma and beta fractions: "Furthermore, using SHAP values also enabled us to compute an M-protein probability score for each patient (Fig. 3C), which can be used to predict the likelihood of M-protein being present for a specific patient"

• What would be the difference with predicting the probability instead of the label, using the sklearn package?

While predicting probabilities can provide valuable information about the model's confidence in its predictions, it introduces the need for setting custom decision thresholds (in order to ascert membership in a class or not). In a clinical setting, this can complicate and delay the diagnostic process and may not be well-received by practitioners. Predicting labels also better aligns with common evaluation metrics for binary classification tasks, such as accuracy, precision, recall, and F1-score. These metrics are well-suited for assessing the model's performance in a general setting.

However, we would like to point out that the computation proposed by the Referee is already provided in Fig. 3C, from the Shapley values. As can be seen in that figure, the probability score for the presence of M-proteins in the top sample is 0.10 while in the bottom sample is 0.89.

23-MAJOR / Pages 10-11, Extra Trees and Random Forest show the best success rate in classifying M-protein isotypes: "For this purpose, we had to exclude the samples with free light chain Mprotein, IgD M-protein, and samples with more than one type of M-protein in our data set, since these samples

reached less than 1% of the total M-protein positive samples and were too few to be properly trained and tested by the algorithms."

• I understand the decision of the authors to keep negative samples (i.e., without M-protein) in the training set to keep a robust sample size and increase the trainability of their models. Even though I think it would have been best to train those models with only positive samples (i.e., a dual model pipeline, the first detecting a M-protein and the second classifying it) in order to maintain a high specialization of each model (use the power of two weaker classifiers to make a strong & robust ensemble classifier).

• However, I believe that presenting results including those normal=negative samples leads to biased metrics, since negative samples are the most represented samples and at the same time the easiest to classify (no M-protein = no risk of misclassifying the M-protein).

We acknowledge your suggestion of a dual model pipeline approach, which involves training a model to detect the presence of M-protein and a separate model for classification. We agree that this approach has merits and could lead to specialization of each model. We have in fact carried out such a training and produced the following results but decided to not complicate the exposition further by introducing two models to be carried out in succession instead of one:

	precision	recall	f1-score	support
0	0.96	0.99	0.98	5240
1	0.98	0.86	0.92	1465
accuracy			0.97	6705
macro avg	0.97	0.93	0.95	6705
weighted avg	0.97	0.97	0.97	6705

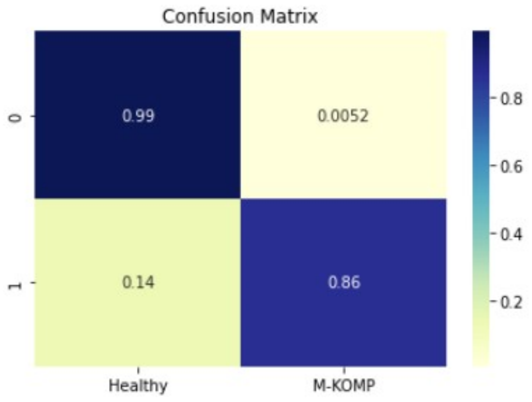

Our primary objective was to build a single unified model for the efficient classification of M-protein isotypes, which we believe can be achieved through feature learning and ensemble methods. We opted for this approach to simplify the model architecture and interpretation, but we understand the trade-offs involved.

We understand your concern regarding the potential bias introduced by the inclusion of negative samples in the training set. It is indeed crucial to consider the impact of class imbalance on our metrics. We have taken measures to account for this bias in our evaluation metrics, such as using precision-recall curves and F1-score in addition to accuracy.

24-MAJOR / Page 11, M-protein detection by decision tree methods can be verified to the gamma and beta fractions:

• The authors show that their models quite accurately classify IgG, less accurately IgA, and even less accurately IgM.

• This would be expected by a “random guest” from an expert, since: 1) IgG are the most common, and commonly found in gammaglobulins; 2) IgM also elude in the gammaglobulins fraction, but are heavier than IgG, so usually responsible of taller M-spikes in the gammaglobulins fraction; 3) IgA are more rare than IgG, but most commonly found in betaglobulins

• How do the authors compare their results to such a “random guest”? For instance, it would be possible to

o 1) make a simple expert system that would systematically classify betaglobulins M-spikes as IgA, and gammaglobulins spikes as IgG or IgM according to the size of the M-spike (by using a threshold determined using the training set)

o 2) compare the trained models to this simple expert system to see if the proposed method outperforms it

• At the very least, this point should be addressed in the Discussion section.

Our models are not meant to excel over an expert’s evaluation of the data. Rather, we are providing a decision support tool for the expert. Thus, indeed, a “random guess” from an expert would be expected to provide similar results as ours.

We view our model as a complementary tool that can be used for example in conjunction with established algorithms designed for immunofixation image analysis, such as the one reported by Hu et al (as referenced on page 20 of our manuscript). In our discussion, on page 19 of the manuscript, we further clarify this application of our model.

We thank the Referee for the proposed instructions on how to create a random guess model. We encounter, however, a serious difficulty in constructing the proposed system. Mainly that of identifying the range (in the x-axis) over which the IgM and IgG M-proteins would appear for each sample. These appear at different locations over the feature space (between 0 and 300) for each sample, and it would be impossible for a computer algorithm to figure this out unsupervised. We envision that this would be possible however if we had an expert annotate a number of samples. If indeed we had the above expertly annotated regions then it would be possible to create threshold values for the M-spikes, over those regions, using the training set and subsequently compare against our current results.

Again, we appreciate the valuable recommendation by the Referee and hope that such a comparison can be made possible in the future.

25-MINOR / Page 11, Discussion: “Analysis of fractionated serum proteins by electrophoresis is the gold standard to evaluate acute phase reactions and immunoglobulin patterns in the individual to help form a diagnosis.”

• Consider revising this sentence which is not entirely accurate: 1) SPEP is a screening method rather than a diagnosis method, which necessitates a confirmation by another technique such as immunofixation; which is a gold standard technique; and 2) acute phase reactions’ gold standard technique would more likely be considered to be plasma CRP, to my knowledge.

The sentence has now been revised as requested.

26-MINOR / Page 11, Discussion: “due to excessive amounts of M-protein”

• Remove “excessive amounts” (the sole presence of a M-protein is, by definition, pathological even without clinical significance)

“Excessive amount” has been replaced by “presence”.

27-MINOR / Page 11, Discussion:

• References 1, 3 and 4 are, in my opinion, inappropriate here, as already discussed earlier.

References 3 and 4 have been removed from the Discussion section. Reference 1 is kept in this section but additional references have been added according to the reviewer’s request.

28-MINOR / Page 11, Discussion: “The discrepancy in accuracy score between our model and the model by Chabrun et al is likely attributable to the fact that different methods were used, but also highlights issues that can arise for networks of high complex nature”

• I believe the origin of this difference is most likely to be attributable to the fact that Chabrun et al. had less precise annotations, since they only performed SPEP. Thus, some samples were classified as M-spike, while some were classified as “restricted heterogeneity of gammaglobulins” (i.e., suspicion of abnormal pattern without clearly defined M-spike). They have, in their study, highlighted than even human experts do not clearly define and agree, for some samples, between normal samples, restricted heterogeneity of gammaglobulins and M-spikes.

• Here, the authors present extremely robust results, which, I believe, were obtained at least in part thanks to clearly defined, binary labels, which were possible thanks to the use of multiple assays (capillary SPEP, gel SPEP, immunofixation). I think the authors should better emphasize this, as it add significant value to their work compare to previous works in the field.

We thank the reviewer for this comment and have extended this part of the discussion to emphasize the robustness of our data.

• However, I think that the sentence “but also highlights issues that can arise for networks of high complex nature” should be discarded: in this context, it has no supportive arguments, and is more likely to reflect the authors’ preference for tree classifiers rather than a solid argument towards the performance of those models compared to ANN/CNN, since those have shown far superior performance in many applications found in literature.

We have removed the sentence as requested.

29-MINOR / Page 13, Discussion: “The use of decision tree methods can be more transparent compared to deep learning algorithms, if implemented as we propose here”

• I think there is too few arguments supporting the fact that tree classifiers (particularly extremely complex models such as the ones trained here) are more transparent than DL classifiers here. The use of the SHAP values seems to be the most recurring argument, however it is a model-agnostic method and thus applicable to ANN/CNN similarly.

Indeed, we agree with the Referee that for a few years now it has also been possible to perform SHAP analysis for CNNs as well and in that respect the use of decision tree is not more transparent than using deep learning algorithms. We have therefore removed this sentence and instead included that "Utilizing decision tree methods offers an effective approach for tackling class imbalance concerns within the data set. Decision trees excel in managing imbalanced data by their inherent ability to prioritize minority classes, thanks to impurity-based splitting criteria like the Gini index or entropy or ensemble methods like Random Forests or gradient boosting, when dealing with several class imbalances."

Reviewer #2

This is a very interesting paper with a clear clinical question (The major clinical question is the presence of monoclonal fraction(s) of antibodies (M-protein/paraprotein), which is essential for the diagnosis and follow-up of hematological diseases, such as multiple myeloma) . They also have a very nice dataset.

1. Regarding the adopted evaluation procedures, there is an important step that is missing: how did the authors choose the hyperparameters? Usually , one performs a gridsearch using k-fold in the training set as GridSeachCV form sk-learn does. If you use the test set to determine the best set of hyperparameters, the results woulb be biased.

The hyperparameters were kept at their default values. In this article we did not try to find the optimal possible values for those hyperparameters but instead focused on finding out the capabilities in terms of performance of these decision tree approaches in learning features from the available data, and, furthermore, pointing out (via SHAP) what they learned in that data. We have now made a comment in the manuscript to clarify the fact that the default values of the approaches are used in the implementations:
"The hyperparameters for all algorithms implemented in this work are set to their default values. These values can be found in the supplementary materials and methods section of the manuscript."

2. it would be nice to compare the results provided by SHAP with standard feature importance that several tree-based algorithms can provide.

We compared the SHAP (for Extra Trees) with a classic feature importance approach as suggested, see below. It is clear from these results that the range of the 10 most important features is between 248 and 259 thus missing the beta fraction. In contrast SHAP identified the range of the 10 most important features from 242 until 260 which includes both the beta and gamma fractions. We have now included these results as supplementary figure S4 in the manuscript.

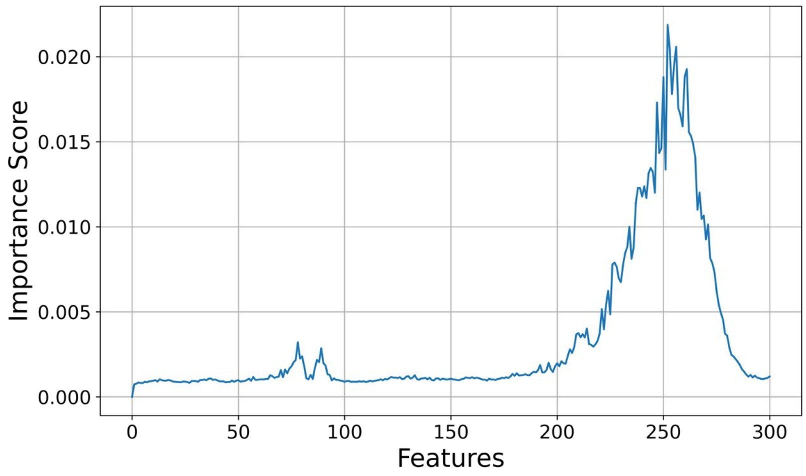

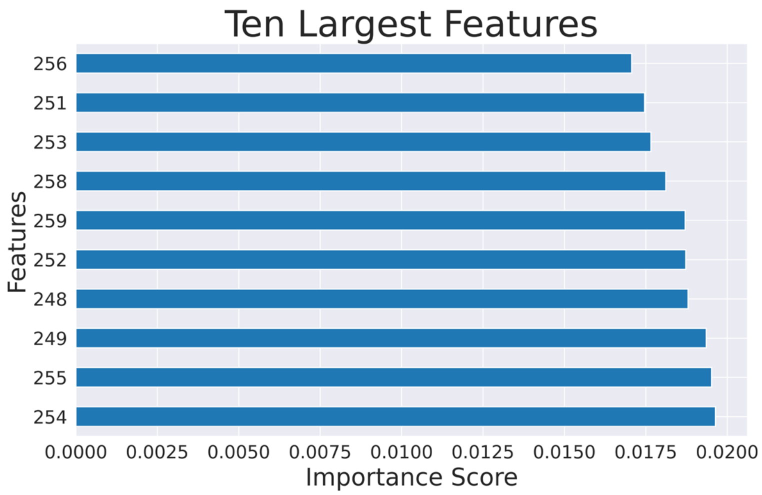

3. In the discussion , the authors say “Deep learning algorithms are highly suitable and powerful for image analysis but exhibit some disadvantages. One such weakness is the “black box” phenomenon, the

inability to explain how the outcome result was achieved”. One can say the same thing about XGB or LGBM. For deep learning , there are several explanation methods that could be employed, including SHAP. Please correct this statement.

The Referee is correct. Originally it was mainly decision tree based algorithms that were capable of producing information about feature importance. It has since become possible for most types of neural networks, including deep networks as well as XGM and LGMB, to produce similar results. We have now replaced the original text in the manuscript with the following: "Complex machine learning algorithms, including deep learning models, as well as methods like XGBoost and LightGBM, share a common limitation referred to as the 'black box' phenomenon, where understanding the internal decision-making process leading to outcomes becomes challenging. Explanation methods, such as SHAP, can be employed to enhance the interpretability of these models by providing insights into feature importance and contributing factors."

4. In the discussion the authors also say “The use of decision tree methods can be more transparent compared to deep learning algorithms, if implemented as we propose here, but work best for numerical series of data rather than data retrieved from images”. Decision trees are more transparent, but this is not true for the other algorithms derived from them as XGB. It is also very hard to tune XGB or LGBM hyperparameters, because there are several of them(see the documentation)

We appreciate the Referee's comments regarding the transparency of decision tree methods and the derived algorithms like XGBoost (XGB) and LightGBM (LGBM). We agree that while decision trees are generally more transparent due to their rule-based nature, the same level of transparency might not directly translate to more complex models like XGBoost and LightGBM. We have now revised that statement in the discussion to the following: "While decision tree methods can offer increased transparency due to their rule-based nature, it's important to note that this transparency might diminish as we move to more complex algorithms like ensemble models including XGBoost (XGB) and LightGBM (LGBM). These ensemble methods can introduce additional complexity that could impact the interpretability of the models and can make it harder to identify suitable values of their hyperparameters."

5. The authors say that the limitation of their approach was the relatively low accuracy scores for identifying the specific isotype of M-protein present, probably due to the low number of certain M-protein isotypes in the population. What the authors had was an imbalanced dataset. This could be correct using SMOTE techniques.

Indeed, as the Referee points out, we have an imbalanced dataset and we could have chosen to carry out a number of approaches to correct this issue such as a resampling technique (minority over sampling as the Referee suggested) or even an algorithmic approach (cost-sensitive learning), but in the end all of these would be open to criticism which we decided to avoid. We believe however it is a good point to bring up in the discussion and we have now added a comment as follows:
"In the context of identifying specific M-protein isotypes, it is important to consider the potential impact of class imbalance within the dataset. The relatively lower accuracy scores observed in our approach may be attributed to the presence of certain M-protein isotypes that are inherently less prevalent within the population. This imbalance in class distribution can pose challenges for accurate classification. To mitigate this issue and enhance the model's ability to discern rarer isotypes, techniques such as SMOTE (Synthetic Minority Over-sampling Technique) could be explored. By generating synthetic instances of underrepresented isotypes, SMOTE aims to balance class proportions and improve model performance.

The incorporation of such methods could help address the impact of class imbalance and potentially lead to more accurate identification of specific M-protein isotypes."
