# Supplementary material for: Machine learning evaluation for identification of M-proteins in human serum
Source: PLoS One. 2024 Apr 2;19(4):e0299600. doi: 10.1371/journal.pone.0299600 (PMC10986985; doi:10.1371/journal.pone.0299600)
Supplement: Attachment — Submitted filename: Response to reviewers.pdf [file pone.0299600.s008.pdf]

Reviewer #1: First, I want to thank the authors for carefully studying and replying to each and every one of my comments, and for the thorough work I believed they have undertaken for improving their manuscript. In my opinion, all major concerns regarding this study have been addressed.

Particularly, the current version of their manuscript now enables a detailed understanding of the exact data used by the authors and potentially accessible to the community, notably due to the addition of Tables 1 and 2 and confirmation that all samples were double checked with both CZE and gel SPEP. This confirms the high value of those data.

We thank the reviewer for carefully and thoroughly assessing our manuscript and our changes and responses in the first round of revision. We have addressed the new comments as seen below in a point-by-point response. We hope that our responses have resolved any remaining uncertainties that the reviewer had.

I still have a few minor comments to the authors, which may or may not trigger modifications in the final manuscript, to their discretion:

- New tables 1 and 2 are interesting and I feel are nice additions to the manuscript. In Table 2, I am not sure about the use of the word “tertiles”, which implies that selected cut-offs, namely 1g/L and 5g/L, would divide the dataset into three subsets of equal size (1 third of the samples), which is not the case here.

We have removed the word “tertile” from Table 2 as requested and instead added “*three groups: <1 g/l, 1-5 g/l and >5 g/l*”.

- Several arguments against the use of deep learning in this study seem fallacious in my opinion. For instance:

o the assumed ability of tree models to better handle class imbalance due to Gini index/entropy overlooks the fact that cross entropy is one of the most widely used loss functions in deep learning; for the very same reason.

o the authors imply that convolutional layers would not be suitable to the analysis of 306-point-wide traces reshaped to 17x18 images. Analyzing small images is perfectly performed by CNN, for instance on countless MNIST examples online (28x28 images). Furthermore, 306-wide traces can (and should here) be reshaped to (306,1) arrays/tensors to avoid jeopardizing spatial information, as described in several previous works analyzing 1-dimensional signal. Finally, authors could even use 1d-conv layers to avoid reshaping raw traces if reshaping itself is a concern.

o I can understand that the authors refuse to use DL compared to tree models, and I think the “no free lunch theorem” sufficiently justifies this strategy. But in my opinion, the specific arguments cited above are misleading and should be removed from the final version.

At the reviewer’s request, we have now removed the paragraph with our arguments against deep learning methods in our setting from the discussion.

- Authors corrected the name of the python package they used from “shapely” to “Shapely” version 1.7.1. I would like to make sure this is not a mistake: Figure 3 highly resembles plots obtained with the “SHAP” package based on “ShapLEy” (not “ShapELy”) values. Furthermore, SHAP is also cited multiple times by the author. ShapELy v 1.7.1 seems to be a Python package related to geometric analysis rather than feature importance. Can the authors confirm there is no typo here?

We thank the reviewer for correction. Indeed, we are using the SHAP (SHapley Additive exPLanations) python library package to perform feature importance analysis and not the Shapely 1.7.1 library. This typo is now corrected in the manuscript. The following incorrect text "*Feature importance was based on the Shapely values from the Shapely 1.7.1 library*" has been replaced by "*Feature importance was based on the Shapley values from the python package shap version 0.37.0*”.

- Knowing that the authors, even unknowingly, complied with recommendations such as STARD is extremely positive. I think the readership’s trust would highly benefit from citing this in the Methods section, though this is not mandatory.

We thank the reviewer for the suggestion. A citation has now been added to the Materials and Methods section that states that our study aligns with the STARD recommendations for reporting diagnostic studies.

- The table cited in the “Response to reviewers” file, depicting the performance of models according to the M-spike concentration (<1g/L, 1-5g/L, >5g/L) is really interesting, and in my opinion its addition in the manuscript or supplementary material, or at least a sentence in the Results would be of interest for the readers of this work.

We have added a sentence in the Results section as requested.

Reviewer #3: This is an interesting study that demonstrates the analysis of a large set of electrophoresis data using machine learning for the identification of M-proteins in serum. Two referees have thoroughly assessed the paper and provided useful feedback which has been carefully addressed by the authors. While I agree that the ML algorithms used are not overly sophisticated, their applicability to the problem makes the procedure easy to implement. It would be good if this could be tested on 'new' electrophoresis data from various sources to test the transferability of the method, but I am aware this is beyond the scope of the current study. The dataset is extensive and relevant and I think the paper will make a good addition to the literature.

We thank the reviewer for carefully and thoroughly assessing our manuscript and our changes and responses in the first round of revision. We are very grateful that Reviewer #3 took on the task as a new reviewer and appreciate the work the reviewer put in as well as the comments. We hope to access more electrophoresis data in the future from other sources to further test our method as suggested, but as the reviewer already pointed out, this is beyond the scope of the current study.
